# Reduction rate by decompression as a treatment of odontogenic cysts

**DOI:** 10.4317/medoral.21916

**Published:** 2017-08-16

**Authors:** Luis Oliveros-Lopez, Ana Fernandez-Olavarria, Daniel Torres-Lagares, Maria-Angeles Serrera-Figallo, Raquel Castillo-Oyagüe, Juan-Jose Segura-Egea, Jose-Luis Gutierrez-Perez

**Affiliations:** 1 DDS. School of Dentistry. University of Seville; 2 PhD, DDS, MSc (Oral Surgery). Professor of Oral Surgery. Department of Stomatology. University of Seville; 3 PhD, DDS. School of Dentistry. University of Seville; 4 DDS, PhD. School of Dentistry. University Complutense of Madrid; 5 DMD, PhD. Professor of Conservative Dentistry. Chairman of Conservative Dentistry. Department of Stomatology. University of Seville; 6 MD, DMD, PhD. Professor of Oral Surgery. Chairman of Oral Surgery. Department of Stomatology. University of Seville

## Abstract

**Background:**

Odontogenic cysts are defined as those cysts that arise from odontogenic epithelium and occur in the tooth-bearing regions of the jaws. Cystectomy, marsupialization or decompression of odontogenic cyst are treatment approach to this pathology. The aim of this study was to evaluate the effectiveness of the decompression as the primary treatment of the cystic lesions of the jaws and them reduction rates involving different factors.

**Material and Methods:**

23 patients with odontogenic cysts of the jaws, previously diagnosed by anatomical histopathology (follicular cysts (7) and radicular cysts (16)) underwent decompression as an initial treatment. Clinical examination and pre and post panoramic radiograph were measured and analyzed. In addition, data as gender, age, time reduction and location of the lesion were collected.

**Results:**

Significant results were obtained in relation to the location of lesions and the reduction rate (*p*<0.01). In a higher initial lesion, a greater reduction rate was observed (*p*<0.05).

**Conclusions:**

Decompression as an initial treatment of cystic lesions of the jaws was effective; it reduces the size of the lesions avoiding a possible damage to adjacent structures. Cystic lesions in the mandible, regardless of the area where they occur will have a higher reduction rate if it is compared with the maxilla. Similar behavior was identified in large lesions compared to smaller.

** Key words:**Decompression, reduction rate, cyst, maxilla, mandible.

## Introduction

Odontogenic cysts are defined as those cysts that arise from odontogenic epithelium and occur in the tooth-bearing regions of the jaws. It is usually considered that proliferation and/or degeneration of this epithelium leads to odontogenic cyst development. Cystic jaw lesions may be epithelial or non-epithelial, odontogenic or non-odontogenic, developmental, or inflammatory in origin (1-3).

The growth of cystic lesions in the jaw can cause damage to adjacent vital structures such as the mandibular nerve and maxillary sinuses. They also can cause facial asymmetry, dental displacements and pathological fractures. To avoid this, various treatments have been described for handling the jaw cysts (4,5).

The marsupialization, was described by Partsch in 1892 (6), it is a technique where a large window in the cyst wall is made and then sutured to the oral mucosa. This communication path between the oral cavity and the cyst will decrease the internal pressure of the lesion and will promote the generation of new bone (6-10).

Decompression of odontogenic cyst can be performed with the use of various devices (tube, stent) and it consists in make a small window in the lesion to subsequently suture the tube on its periphery (11). The histological changes in the cystic capsule are much more discreet if it is compared with the changes on the marsupialization (4) This procedure eliminates the intramural pressure, activating the bone formation within the lesion. This treatment requires a monitoring and commitment by the patient (12,13).

Cystectomy is the complete enucleation of the lesion; is a more aggressive and fast technique but it has the risk of compromising important structures nearby (4,6,7).

In such cases, when these risk situations have been detected, an initial approach with decompression, followed by cystectomy, may be appropriate (4,14).

Different authors have written the effectiveness of decompression. Anavi and colleagues in 2011 described decompression in 73 patients, as effective in reducing odontogenic cysts (13). Then Gao and colleagues in 2014 described decompression as a treatment that reduces radicular cysts, keratocysts and ameloblastomas, increasing bone density (12).

There is limited data of how the cystic lesions of the jaws can evolve after a decompression and factors influencing its reduction. Therefore, the objective of this study is to evaluate the reduction rate in cyst decompression in a case series of 23 patients with regard to factors such as the location, the initial size, gender or age of the patients.

## Material and Methods

- Study subjects

A retrospective study was performed using the database of patients of Oral Surgery Master at the University of Seville including patients treated with cystic decompression and cystectomy as a final treatment, between February 2008 and February 2015. We selected 23 patients (14 male and 9 female) with 16 radicular cysts (7 female, 9 male), 7 follicular cysts/dentigerous cysts (5 male, 2 female). The age of the patients ranged between 61 and 15 years old. A total of 9 maxillar cysts and 14 mandibular cysts were analyzed. The distribution was 23% (n = 6) in the anterior maxilla, 13% (n = 3) in the posterior maxilla, 9% (n = 2) in the anterior mandible and 55% (n = 12) in the posterior mandible area.

In this period, we identified 683 patients diagnosed as cysts of the jaws. The majority was treated with cystectomy, either as initial intervention (603 patients) or as re-cystectomy after recurrence (52 patients) and therefore excluded from this study. We identified 28 patients treated with decompression and subsequent cystectomy, excluding 5 patients for lack of adequate clinical and radiological data to incorporate them into this study.

- Treatment protocol

The study protocol was approved by the Ethics Committe of the Virgen del Rocio Hospital and of the University of Seville. All patients read and signed the informed consent participation of the study and development for the guidelines outlined in the Declaration of Helsinki for human experimentation.

The indication of the decompression was performed in the presence of a diagnosis of maxillary cyst in relation to a noble area that could be damaged during the cystectomy. We planned a decompression in cases of cysts that had a relationship of wide neighborhood to the maxillary sinus, nasal cavity, or the inferior alveolar nerve.

Intervention was performed always under local anesthesia, by blocking terminal nerves in the area. Using a full thickness flap, vertical (releasing) incisions mesial and distal to the point that will be the decompression tube, leaving a margin of safety of 0.5 cm between releasing incisions and the planned area of bone window. Obviously, noble nearby structures such as the mental nerve was preserved. Once we have access to the bone surface, must do a small window on the cystic capsule to introduce the decompression tube.

An incisional biopsy at the time of decompression was performed for definitive histopathological diagnosis of the lesion. All patients were treated by placing a decompression tube of 4 mm in diameter, which was previously drilled several times and sutured to the oral mucosa for subsequent daily irrigation of the cystic lesion.

Irrigation was performed with 0.12% chlorhexidine mouthwash, two or three times a day, introducing 5 ml of the liquid through the drain tube, using a syringe and cannula non-traumatic, and leaving to the liquid back out after washing the cavity.

**Figure 1:**
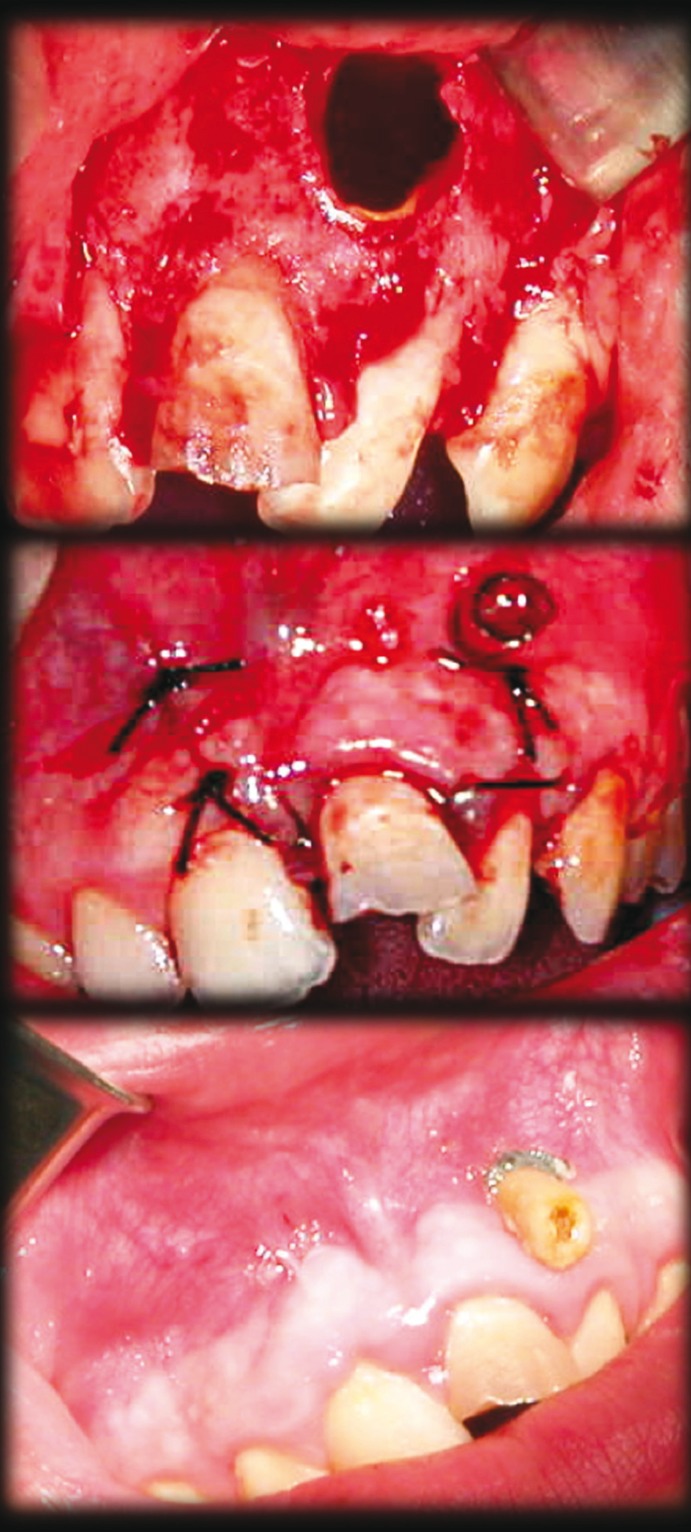
Decompression in one of the cysts. Opening of the cystic capsule and biopsy sampling. Tube placement immediately after surgery. Tube placed during decompression.

Revisions were made to the patient every two months, panoramic radiograph were taken and adjustments to the decompression tube where made, if it required (Fig. 1).

All patients were treated in the Department of Oral and Maxillofacial Surgery at the Hospital Universitario Virgen del Rocío in Seville and in the Master of Oral Surgery at the University of Seville. All diagnostic radiographs (panoramic radiographs) were taken and analyzed with the Romexis program (Planmeca, U.S.A.).

Before the decompression, all patients were informed about the goals of this treatment, explaining everything on a previous panoramic radiograph or tomography.

When the cyst was reduced enough to avoid the adjacent structures, proceed to perform the surgical excision of the lesion using the last panoramic that was taken. In the case of inflammatory cysts, all teeth with negative vitality that were at that time affected by the cystic cavity were treated with root canal treatment before the intervention. During cystectomy, these teeth was treated with apicectomy and surgical retro-filling.

Exclusion criteria for the study were missing clinical or radiographic data, failure to maintain the decompression window, and follow-up less than 6 months.

- Measurements

Radiographs were observed, measured and analyzed by two residents of the Master of Oral Surgery at the University of Seville (LGOL and AFO). All measurements were repeated twice to minimize measurement bias. Inter-examiner reproducibility was calculated (k = 0.975). All radiographs were measured and analyzed using Image J program (National Institutes of Health, U.S.A.). In each patient there was a first measure in radiograph, mesio-distal width of the crown of the closest tooth to the lesion, and a clinical measure of the same tooth. With these measures a full scale of the panoramic was obtained.

**Figure 2:**
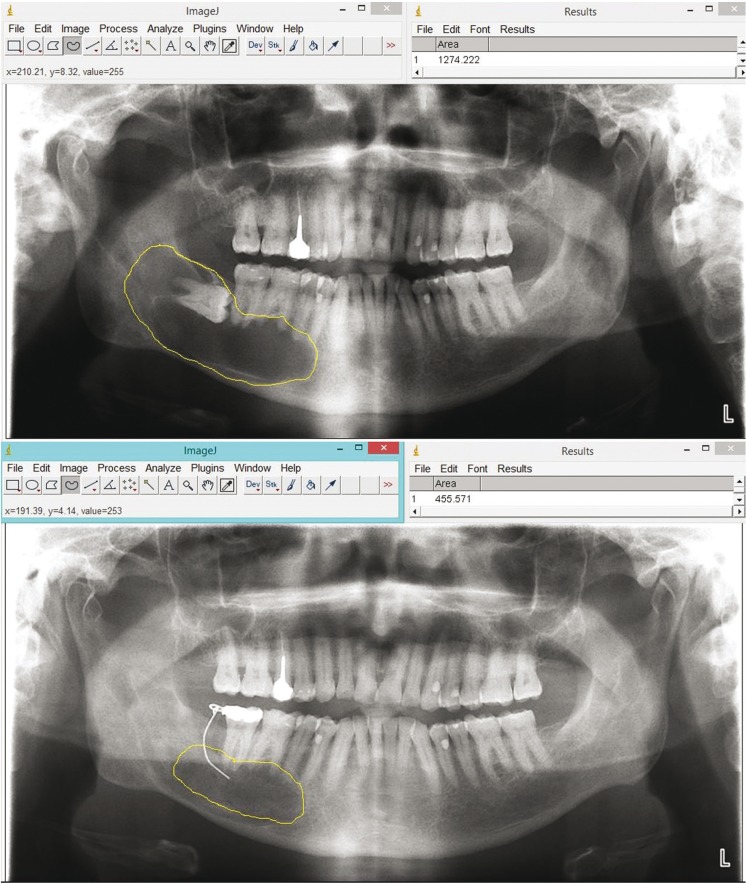
Measurement of initial and final area in a cyst of the simple.

The reduction rate of the size of cysts was calculated as follows: initial area minus the final area expressed in square millimeters (mm2) divided by the time expressed in months observation (Fig. 2).

All panoramic radiographs were taken as follows to avoid errors in measurements and ghost images: the upper and lower incisors were placed in a bite guide, the Frankfort’s plane had to be parallel to the ground, the sagittal plane had to be parallel to the ground and centered on the bite guide, the patient’s back had to be and remain straight, the patient’s tongue must remain in the palate for 20 seconds until the process was complete.

- Statistic analysis

Data were collected using Excel (Microsoft, U.S.A.) and analyzed using SPSS 13.0 (SPSS Inc., U.S.A.). The chi-square was performed to compare the data of the variables between the groups and their distribution.

## Results

A total of 23 patients were treated with cystic decompression of the jaws. The reduction rate was analyzed according to the distribution in the following groups: age, gender, histology, lesion location and the size ( Table 1). The histological type were divided and compared with groups, and reduction rate was analyzed ( Table 2).

**Table 1:**
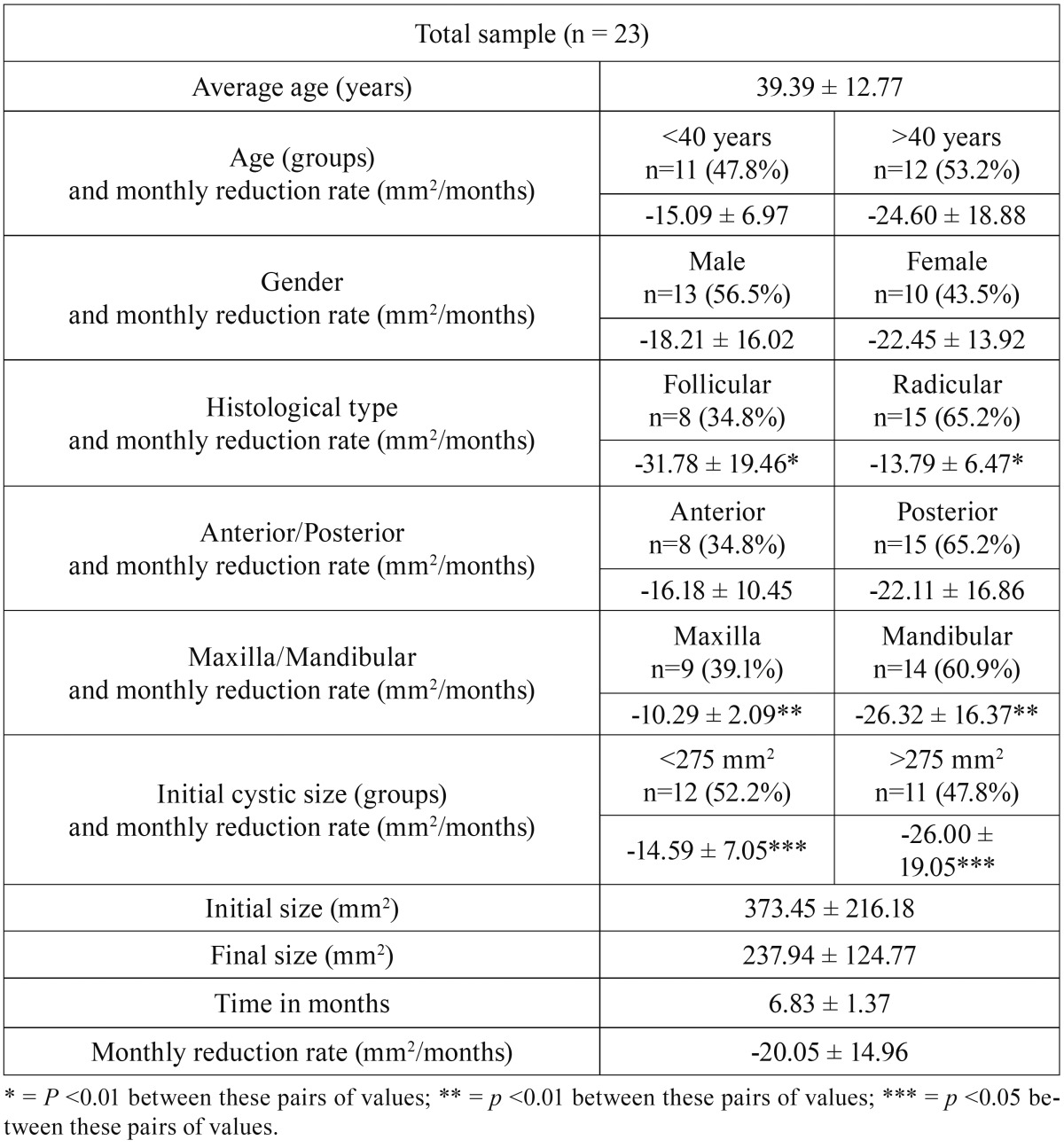
Descriptive data of the sample, as well as values of monthly reduction rate of the cyst among the different subgroups.

**Table 2:**
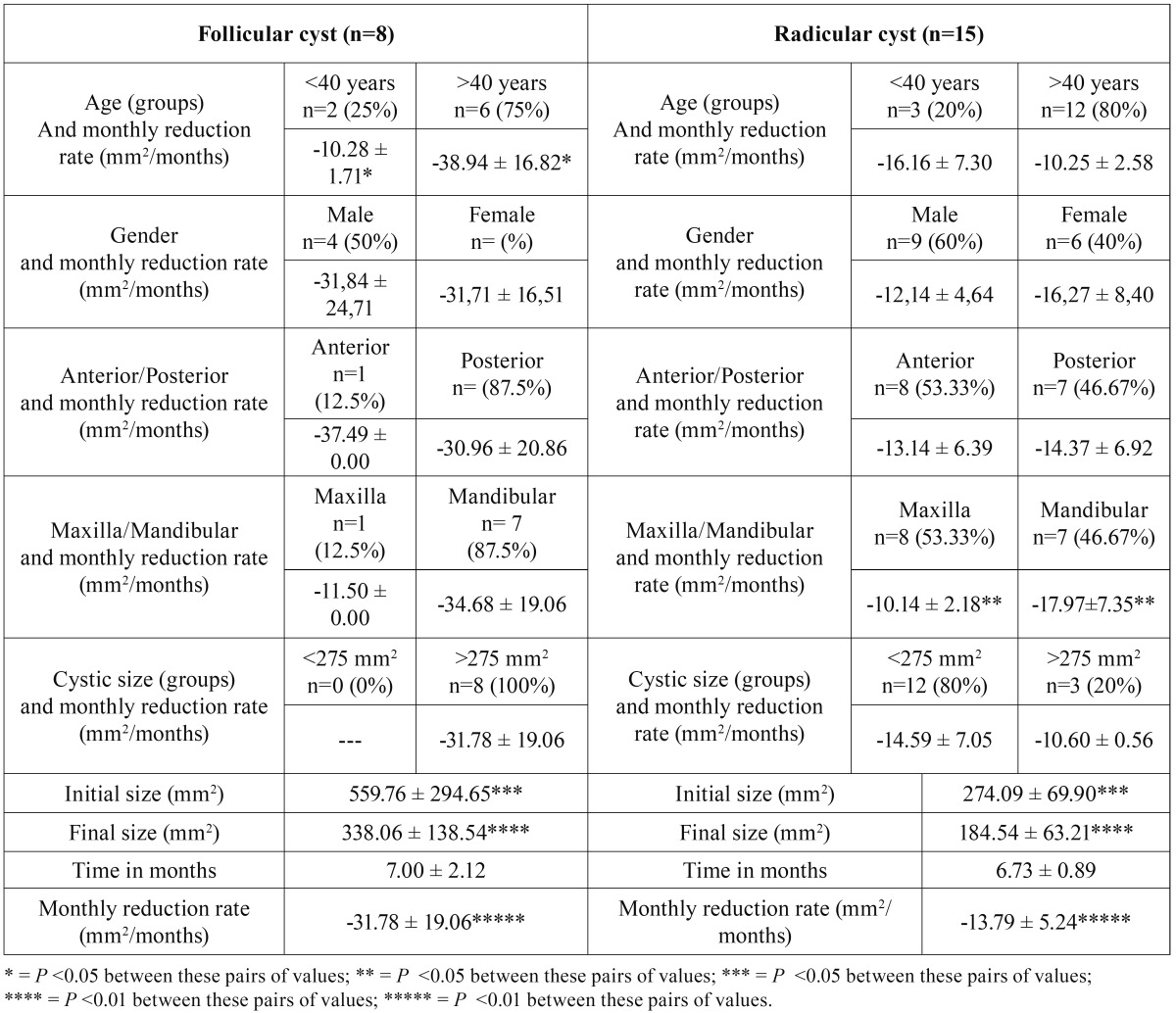
Descriptive data of the sample divided by histological type, as well as values of monthly reduction rate of the cyst among the different subgroups.

The descriptive data was distributed in the following variables: the Maxillary-mandibular position; Anterior-posterior position; Cyst size and histological type, as the significance of associated chi-square ( Table 3).

**Table 3:**
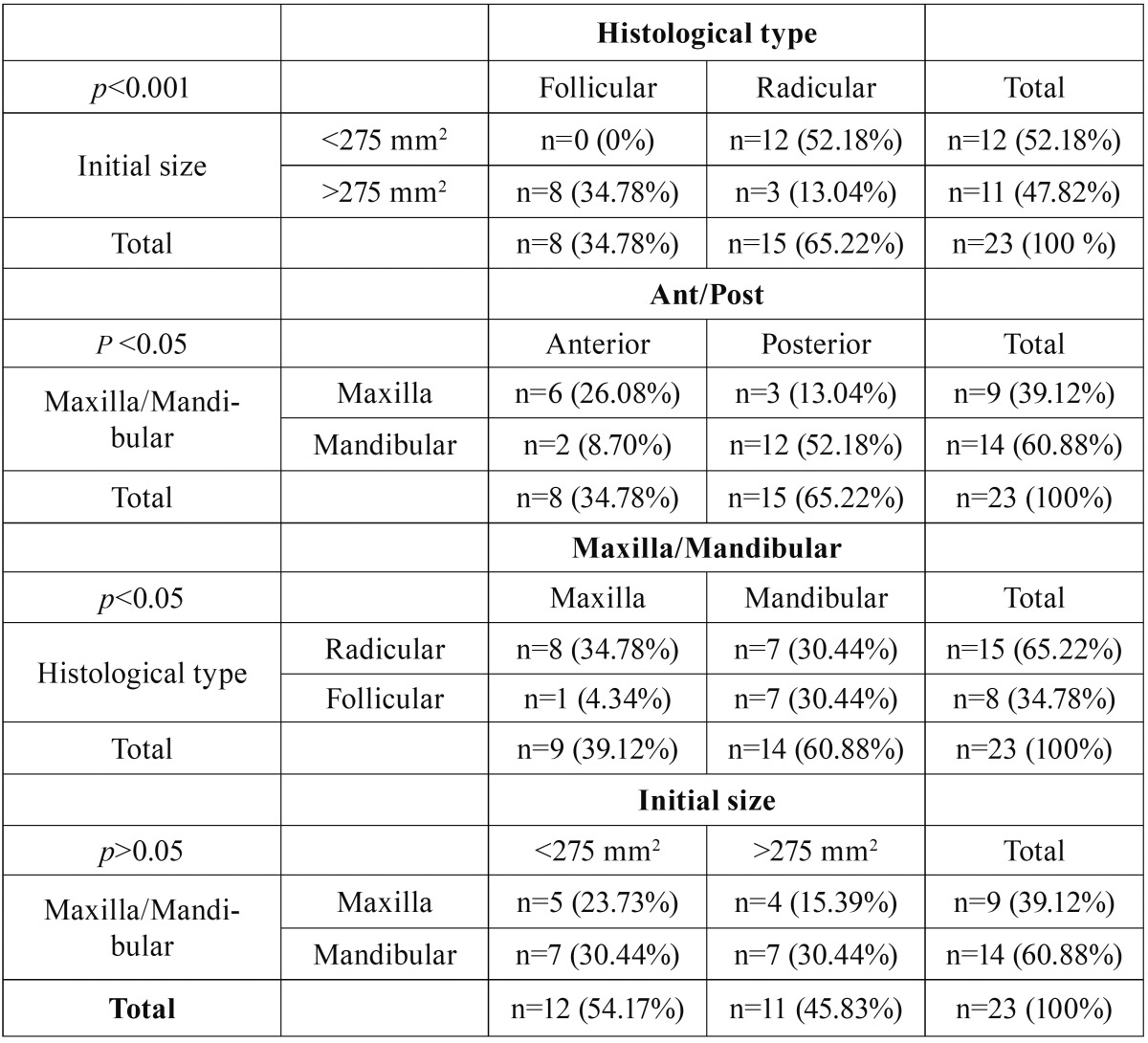
Descriptive data of the distribution in tables 2x2 of the following variables: the Maxillary-mandibular position; Anterior-posterior position; Cyst size and histological type, as the significance of associated chi-square.

Considering the histologic type, we obtained a monthly average reduction of -31.78 ± 19.46 mm2 in dentigerous cystic lesions and -13.79 ± 6.47 mm2 in radicular cysts with statistically significant difference was found (*p*<0.01).

In the location of the lesion we obtained more reduction rate in the mandible -26.32 ± 16.37 and -10.29 ± 2.09 in the maxilla with statistically significant differences were found (*p*<0.01).

The monthly reduction rate was better in lesions greater than 275 mm2 with -26.00 ± 19.05 and -14.59 ± 7.05 in lesions lower than 275mm2 with statistical significant difference (*p*<0.05).

In the follicular histologic type we had that in patients younger than 40 years the monthly reduction rate was -10.28 ± 1.71 and -38.94 ± 16.82 in patients older than 40 years, with a significant statistical difference (*p*<0.05). In the radicular cysts we obtained that in patients younger than 40 years the reduction rate was -16.16 ± 7.30 and -10.25 ± 2.58 in older than 40 years.

In the radicular histologic type we had better reduction rate in the mandible with -17.97 ± 7.35 and -10.14 ± 2.18 in the maxilla, with significant statistical difference (*p*<0.05).

In the initial size of follicular lesions we found significant statistical difference (*p*<0.05) in monthly reduction rate with 559.76 ± 294.65 versus 274.09 ± 69.90 of radicular histologic type. We observed similar behavior in final size of follicular cysts, with 338.06 ± 138.54 versus 184.54 ± 63.21 in radicular cysts, with statistical significant difference (*p*<0.05).

The monthly reduction rate was faster in follicular cysts with -31.78 ± 19.06 versus -13.79 ± 5.24 in radicular cystic lesions, statistical significant difference (*p*<0.01). We had not observed statistical significant difference between monthly reduction rate on gender and anterior-posterior lesions of the jaws.

When we obtained statistically significant differences in the histological type, and try to compare it with the size of the cysts and their location ( Table 3), we noticed that such comparison was not possible, so we elaborated a comparing cysts table with the same histology, same location and similar sizes ( Table 4).

**Table 4:**
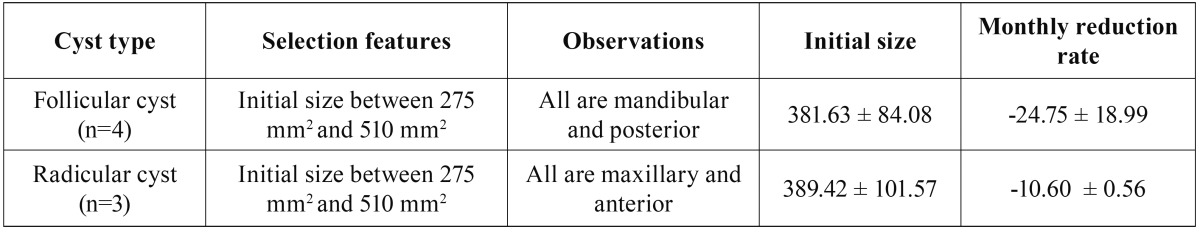
Initial size and monthly rate of descent of a subsample of follicular.

We found that in the follicular cysts (4) with initial size between 275mm2 and 510mm2 located in posterior mandibular zones, the monthly reduction rate was greater than in the radicular cysts (3) with the same sizes and located in the anterior maxilla.

## Discussion

Several treatments have been described for the management of maxillary and mandibular cysts, although none has been accepted globally (15,16). Nowadays, decompression, marsupialization, enucleation and resection of lesions are accepted as valid (4,12,14).

The benefits of marsupialization and decompression include the gradually decreasing the cystic cavity; preserving the adjacent oral tissues, maintaining pulp vitality, avoiding dental extractions, preventing iatrogenic damage to adjacent noble structures, avoiding mandibular fractures and reducing the risk of recurrence (9,10,13-15). In all cases, a second surgery is needed to elimi-nate totally the cystic lesion after decompression (4).

Decompression and marsupialization are procedures that require patient’s commitment. They need several control appointments and constant hygiene with repeated irrigation, gauze soaked in iodine of the cystic cavity. One of the main disadvantages of marsupialisation is communication between the cyst and oral cavity which may facilitate infection of the lesion.

Among the principal disadvantage of decompression we have the tube lost, the obliteration of the tube’s entrance, difficulties to rinse off, problems wuth irrigation and infections.

Anavi *et al.* in 2011, performed decompression in odontogenic cysts with subsequent cystectomy. In 60% of cases there was a good ossification of the area after decompression. In the present study we observed a correct ossification of the area after decompression and then we proceed to make the enucleation of the lesion (13).

Enislidis *et al.*, in 2004, made a study in large cysts, which reported that there was no recurrence after the treatment of decompression with subsequent cystectomy. These results concurred to the present study, where recurrence was not observed after one or two years follows-up of treated patients. This leads us to think about the high predictability of this treatment (15).

Anavi *et al.* (13) and Asutay *et al.* (18) did not obtain statistically significant differences in the reduction rate depending on gender, coinciding with this study.

In the present study we showed that greater reduction rate occurs in large lesions. This agrees with the studies of Gao *et al.* (12) and Park *et al.*(16) where speed of decompression in extensive cystic lesions was analysed.

Lizio et al. (13) Kubota *et al.* (17) and Gao et al. (12) did not find relation between reduction rate and patient´s age, as this study did. However, Anavi et al., (13) Park *et al.*, (16) Asutay *et al.* (18) and Song *et al.* (19) found relation between age and reduction rate.

Song *et al.* (19) and Asutay *et al.* (18) studied the descompression on the following final histologic diagnosis: dentigerous cysts, ameloblastomas and odontogenic keratocysts. This study was performed using radicular cysts and dentigerous cysts so we considered as a limitation.

Anavi et al. (13) studied the cystic reduction rate with the following histologic diagnosis: dentigerous cysts, odontogenic keratocysts, radicular cysts and glandular odontogenic cyst, so we could compared some of our results with these group. They did not obtained statistical significant diferences between pathological type. It disagrees with the results of this study where the monthly reduction rate was faster in dentigerous cysts with -31.78 ± 19.06 versus -13.79 ± 5.24 in radicular cystic lesions (*p*<0.01).

Anavi *et al.*, (13) show that decompression is faster in patients under 18 than it is in patients older than 18 years old. Asutay *et al.* (18) and Song *et al.* (19) describe that older patients, have smaller reduction of cystic lesions. This disagree with our study, where a higher reduction rate was observed in older patients. It is considered that this value results from the average age of patients, 40 years old, which leads us to think about the lack of patients younger than 40 years in our study, considered as one of the limitations.

Park *et al.* (16) and Asutay *et al.* (18) describe that decompression as a treatment for cystic lesions should last until the lesion decrease its size and risks of vital structures injury during enucleation dissapear. These opinions agree with those explained in this study.

Rubio *et al.* (20) showed good results after cystectomy without filling the cavity with biomaterials. That coincides with this work, because correct bone fill of the cavities was observed radiographically after one year follow, despite not having made measurements of them.

Song *et al.*, (19) in 2015, performed a study of cyst decompression where the lesions where only located, in body and mandibular ramus and they obtained good results.

According to the location of the lesions, maxilla or mandible, we found statistically significant results (*p*<0.01). It can be concluded that the decompression of mandibular lesions, regardless of the area where they are, would have a higher reduction rate than maxillary cysts. These results are not reflected in any study published until the moment.

Enucleation is still the main choice of treatment for many authors, this procedure involves the complete removal of the lesion with subsequent curettage of the surgical site. The main advantages are not several control visits or special hygiene measures. For large odontogenic cysts a comprehensive evaluation is recommended before performing this treatment, to avoid injury to adjacent noble structures (4).

In our study, we performed all the measurements in panoramic radiographs, therefore they were done in two dimensions. With this, we can have an idea of what lesions measure is, even so we advise the use of 3-dimensional CT to obtain more information about the lesion.

We consider that 23 patients are not enought to establish clear conclusions but it is to establish a hypothesis and create tendencies. We will continue observing the behavior of the dentigerous cyst and radicular cyst in more patients for future studies.

## Conclusions

Decompression, as initial treatment of cysts of the jaws proved to be effective. It reduced the size of lesions avoiding injury to adjacent structures. Cystic lesions in the mandible, regardless of the area where they occur will have a higher reduction rate than maxillary cysts. Similar behavior is identified in large lesions compared to smaller.
